# Acid ceramidase expression reduces IFNγ secretion by mouse CD4^+^ T cells and is crucial for maintaining B-cell numbers in mice

**DOI:** 10.3389/fimmu.2024.1309846

**Published:** 2024-06-11

**Authors:** Putri Mandasari, Claudia Hollmann, Rehan-Haider Zaidi, Samira Löw, Jann Schrama, Dominik Wigger, Fabian Schumacher, Burkhard Kleuser, Niklas Beyersdorf

**Affiliations:** ^1^ Institute for Virology and Immunobiology, University of Würzburg, Würzburg, Germany; ^2^ Institute of Pharmacy, Freie Universität Berlin, Berlin, Germany

**Keywords:** CD4+ T cell, acid ceramidase, cytokine secretion, inducible knockout mice, B cell survival, *in vitro* culture assay

## Abstract

Acid ceramidase (Ac) is a lysosomal enzyme catalyzing the generation of sphingosine from ceramide, and Ac inhibitors are currently being investigated as potential cancer therapeutics. Yet, the role of the Ac in immune responses, particularly anti-viral immunity, is not fully understood. To investigate the impact of Ac expression on various leukocyte populations, we generated a tamoxifen-inducible global knockout mouse model for the Ac (iAc-KO). Following tamoxifen administration to healthy mice, we extracted primary and secondary lymphoid organs from iAc-KO and wild-type (wt) littermates and subsequently performed extensive flow cytometric marker analysis. In addition, we isolated CD4^+^ T cells from the spleen and lymph nodes for sphingolipid profiling and restimulated them *in vitro* with Dynabeads™ Mouse T-activator CD3/CD28. Intracellular cytokine expression (FACS staining) was analyzed and secreted cytokines detected in supernatants. To study cell-intrinsic effects, we established an *in vitro* model for iAc-KO in isolated CD4^+^ T and B cells. For CD4^+^ T cells of iAc-KO versus wt mice, we observed reduced Ac activity, an increased ceramide level, and enhanced secretion of IFNγ upon CD3/CD28 costimulation. Moreover, there was a marked reduction in B cell and plasma cell and blast numbers in iAc-KO compared to wt mice. To study cell-intrinsic effects and in line with the 3R principles, we established *in vitro* cell culture systems for iAc-KO in isolated B and CD4^+^ T cells. Our findings pinpoint to a key role of the Ac in mature B and antibody-secreting cells and in IFNγ secretion by CD4^+^ T cells.

## Introduction

Sphingolipids were first discovered over six decades ago and have been since researched widely to understand their metabolism and functions (1, 2). They are best known as the building blocks of the plasma membrane, and their bioactive properties render them very important in various cellular functions and pathophysiology (2, 3). Moreover, pro-apoptotic ceramide has been established as the hub in the context of sphingolipid metabolism (4). This is because ceramide forms the center of various sphingolipid pathways, i.e., the *de novo*, sphingomyelinase, and the salvage pathway (3). The ceramide-metabolizing enzyme acid ceramidase (Ac) has received much attention in cancer research (5). This is due to the fact that the Ac was found to be aberrantly expressed in various human cancers including prostate cancer, melanoma, acute myeloid leukemia, and glioblastoma (6–9). Interestingly, loss-of-function mutations in the Ac gene (human, *ASAH1*; mouse, *Asah1*) result in a lysosomal storage disorder named Farber granulomatosis or Farber disease (FD) (10). Ceramide accumulation in most tissues, especially the central nervous system, subcutaneous tissues, and the respiratory tract is the hallmark of this disease (11). The classical manifestation of FD includes inflammatory granulomata, subcutaneous nodules, progressive joint deformation, and hoarseness (12). Strikingly, blood and histopathological analyses of FD patients showed elevated leukocyte numbers and enlargement and histiocytic infiltration, i.e., infiltration by foamy histiocytes and macrophages, of different organs including lymphoid tissues (13). FD is a very rare disease with an estimated incidence of less than one in a million meaning that <200 patients have been reported worldwide since the first description of the disease in 1947 (13). In line with this, only the most obvious immunological phenotypes relating to the already mentioned accumulation of histiocytes and macrophages in tissues have been reported.

To study the biological function of the Ac and for future drug development, several pharmacological inhibitors like B13, ceranib-2, and carmofur have been tested mostly in the context of cancer (14–17). Complete knockout of the Ac turned out to be embryonically lethal (18). Therefore, Ac function was first studied in the context of oocyte development in a conditional knockout mouse (19). To mimic FD, another mouse model was generated by introducing a single nucleotide mutation into the *Asah1* locus (P361R/P361R) (20). These mice were viable but had developmental problems from 4 weeks of age onwards showing typical signs of FD including organomegaly and macrophage and neutrophil infiltration into different tissues (20, 21). In line with these phenotypes, they found that the monocyte chemoattractant protein (MCP-1), macrophage inflammatory protein 1a (MIP-1a), and interferon gamma-induced protein 10 (IP-10) were significantly elevated in the sera of FD mice (20–22).

To avoid the pitfalls associated with studying mice with constitutive and global Ac deficiency, conditional and conditional and inducible knockout mice with exon 1 of the *Asah1* gene flanked by loxP sites (23) (brief: Ac-floxed mice) were used to study the role of the Ac in a variety of inflammatory diseases. Constitutive deficiency for the Ac in endothelial cells was, thus, found to lead to vascular hyperinflammation due to increased activation of the NRLP3 inflammasome and elevated release of IL-1β compared to wt controls (24). For macrophages, it has, further, been shown that Ac expression is required to contain herpes simplex virus replication with Ac deficiency leading to overwhelming viral replication in mice *in vivo* (25). In a mouse model of malaria, Ac deficiency led to decreased parasitemia due to impaired erythropoiesis (26). A recent study using Ac-floxed mice together with a T-cell-specific Cre (Ac-floxed-CD4Cre) analyzed the impact of Ac expression in T cells on melanoma progression *in vivo* (27). The authors found that constitutive Ac deficiency in T cells was associated with increased T-cell activation leading to increased accumulation of IFNγ-secreting Th1 cells in tumors and stronger CD8^+^ T-cell-mediated melanoma cell killing (26).

To gain better insights into the contribution of Ac expression to T- and B-cell function under physiological conditions, we generated tamoxifen-inducible global knockout mice for Ac expression (iAc-KO mice). Moreover, we combined *ex vivo*-induced deletion of *Asah1* gene expression with T- or B-cell isolation and subsequent *in vitro* culture to analyze effects of cell-intrinsic Ac deletion.

## Materials and methods

### Mice

C57BL/6J mice bearing an *Asah1fl* mutation (loxP-flanked *Asah1* gene in exon 1) were crossed with homozygous mice containing a conditional Cre-ER^T2^ inserted into the *Rosa26* locus to generate the global knockout mice (iAc-KO). For the global knock experiments, both iAc-KO and wild-type (wt) mice used in this study were homozygous for *Asah1fl* (fl/fl or wt/wt) and heterozygous for Cre-ER^T2^. To generate Ac-specific knockout in regulatory T cells (Foxp3-iAc-KO), C57BL/6J mice obtaining *Asah1fl* mutation were crossed with hemizygous mice harboring a conditional Cre-ER^T2^ knock-in into *Foxp3* locus located in X-chromosome (28). Foxp3-iAc-KO and its wt littermates used in the experiments were hemizygous for the Foxp3-Cre-ER^T2^. For Ac-targeted deletion in CD4^+^ T cells, tamoxifen-inducible CD4-Cre mouse line B6(129X1)-Tg(Cd4-cre/ERT2)11Gnri/J from Jackson Laboratory was purchased and crossed with Ac-floxed mice. Following tamoxifen administration, Cre recombinase will be translocated from cytosol to nucleus, which will lead to exon 1 deletion of the *Asah1* gene.

All mice were bred and housed in the specific pathogen-free animal facility of the Institute for Virology and Immunobiology of Würzburg University in accordance with German law. Animals used for the *in vivo* experiments were aged between 11 weeks and 26 weeks, whereas for the *in vitro* experiments, animals used were between 8 weeks and 43 weeks, which is in line with the 3R principle.

### Tamoxifen preparation and administration

One 40-mg tablet of tamoxifen (Hexal AG) was dissolved in water freshly before *in vivo* per oral administration (p.o.) to achieve the concentration of 12.5 mg/ml. Each animal obtained 1.25 mg in 100 µl tamoxifen suspension for four consecutive days (0.2 mg/g body weight). Tamoxifen administration was adjusted to the body weight when it exceeded 10% increase or decrease in body weight.

To induce expression loss of *Asah1 in vitro*, active metabolized form of tamoxifen was used. Here, 4-OH-tamoxifen (4-OH-Tm) with ≥70% Z isomer (Sigma-Aldrich) was reconstituted in ethanol to obtain concentration of 50 mM and then stored at −80°C. Pooled cells from the spleen and lymph nodes were incubated with 10 µM 4-OH-Tm (1 h, 37°C), which were subsequently washed twice with BSS/BSA before further usage.

### Cell isolation from lymphoid organs

To obtain single-cell suspension, the spleen, lymph nodes, thymus, and bone marrow were ground or flushed through 70-µm cell strainer in BSS/BSA and subsequently washed twice with BSS/BSA. For single splenocyte suspension, erythrocytes were lysed through hypo-osmotic shock using the same volume of 1.8% NaCl and water. This step is followed by 10-min incubation on ice before the supernatant containing the cells was washed again with BSS/BSA.

### CD4^+^ T cell isolation and culture

After 4-OH-Tm treatment, pooled cells from the spleen and lymph nodes were subjected to CD4^+^ T-cell purification using MagniSort™ mouse CD4 T-cell enrichment kit (Thermo Fisher) according to the manufacturer’s protocol.

For pre-expansion culture, 2 × 10^6^ CD4^+^ T cells were seeded in the presence of 0.1 µg/ml CD28-Superagonist (CD28-SA, Clone: D665, ExBio) and 10^7^ Pan Mouse IgG Dynabeads™ (Thermo Fisher) per well (48-well plate; Greiner) in the mouse medium, which consists of RPMI 1640 medium supplemented with 10% FCS, 2 mM l-glutamine, 10 mM glutamine, 10 mM HEPES, 50 µM 2-mercaptoethanol, 50 µg/ml streptomycin, and 100 U/ml penicillin. This culture went for 7 days at 37°C and was split 1:2 on days 3 and 5. On day 7, cells were washed and removed from the beads and subsequently seeded at low density (3 × 10^5^ cells/ml) in mouse medium for 3 days at 37°C. Cells were harvested on day 10 and restimulated with different concentrations of mouse T-activator beads (Thermo Fisher), namely, 10^5^ and 10^4^ T-activator beads for optimal and suboptimal stimulation, respectively. For the restimulation culture, 1 × 10^5^ CD4^+^ T cells were cultured together with 10^5^ (1:1 bead to cell ratio) or 10^4^ T-activator beads (1:10 bead to cell ratio) in a 96-well round-bottom plate (Greiner) in mouse medium in replicates at 37°C. As negative control, cells were incubated with 5 × 10^5^ normal mouse Ig (nmIg; Sigma-Aldrich) beads with concentration of 5 µg/ml (end concentration, 1.25 µg/ml). For coating nmIg beads, 10^7^ pan mouse IgG beads were washed with PBS three times and resuspended with 250 µl PBS. Subsequently, 20 µg/ml nmIg was added to the washed beads suspension and incubated on ice for 15 min with every 5 min resuspending in between. Three times washing with PBS were followed after, and nmIg beads was resuspended in mouse medium (end concentration of 5 µg/ml).

The purity of CD4^+^ T cells used in *in vitro* experiments ranged between 70% and 90% directly *ex vivo* and increased to 91%–98% after the pre-expansion culture with the CD28-SA followed by the resting phase. CD4^+^ T cells isolated after *in vivo* treatment of mice with tamoxifen had a purity of 65%–86%.

### B-cell isolation and culture

B cells were isolated using pan mouse B-cell isolation kit (Miltenyi) according to the manufacturer’s protocol. B-cell purity after isolation was > 95%. B cells (2 × 10^6^) were seeded per well into a 48-well plate and incubated at 37°C for 4 days in mouse medium.

### Flow cytometry

Mouse cells were first blocked for FcyII and FcyIII receptor with anti-CD16/CD32 2.4G2 (15 min, 4°C), then stained for extracellular markers (15 min, 4°C). Dead cells were excluded by staining with fixable Viability Dye-eFluor 780 (eBioscience). After that, cells were fixed and permeabilized with Foxp3/Transcription Factor Staining Buffer (eBioscience) according to the manufacturer’s protocol and followed by staining for intracellular markers. Absolute cell number was calculated by multiplying the viable subset of interest (%) with the Trypan-blue-based cell count. All antibodies used for the flow cytometry are listed in **Supplementary Table S1**.

### Intracellular cytokine staining

Before performing intracellular cytokine staining, 1 × 10^5^ CD4^+^ T cells were cultured together with 1 × 10^5^ T-activator beads in the presence of 1 µg/ml brefeldin A (Sigma-Aldrich) in a 96-well round-bottom plate (4 h, 37°C) (Greiner). As positive control, phorbol 12-myristate 13-acetate (PMA, 50 ng/ml) and ionomycin (750 ng/ml) were used, whereas mouse medium was applied as negative control (Sigma-Aldrich). After incubation, cells were harvested and subjected to Fc receptor blocking and extracellular staining for CD4 and fixable Viability Dye. For fixation and permeabilization, 2% Roti-Histofix was freshly diluted from 4% Roti-Histofix (Carl Roth GmbH) with equal volume of PBS. Cells were incubated within 2% Roti-Histofix for (1.5 h, 4°C) and washed twice with permeabilization buffer (eBioscience) before being stained for IFNγ, IL-10 and IL-2 expression overnight at 4°C.

### Cytokine secretion assay

Supernatants from restimulation assay were collected and analyzed for cytokine secretion using Legendplex mouse Th cytokine kit (Biolegend). Samples were tested for IFNγ, IL-10, IL-2, IL-5, IL-17a, and TNF according to the manufacturer’s instructions.

### DNA isolation and PCR

DNA isolation was performed using NucleoSpin Tissue Kit (Macherey-Nagel) following the manufacturer’s protocol and quantified by Nanodrop (Thermo Fisher). PCR was done using 100 ng DNA and three primers (57447flp, 57448flp, and 57445cre) to validate the recombination of *Asah1* as wild-type (482 bp), non-excised *Asah1* (585 bp), and excised *Asah1* (706 bp). As the internal housekeeping gene control, *Gapdh* was included in the PCR and produced 89 bp product. Conventional PCR was done using HOT FirePol DNA Polymerase kit (Solis Biodyne). The sequences (5′–3′) of the used primers are as follows:

57447flp: ACA ACT GTG TAG GAT TCA CGC AAT TCT CC
57448flp: TCG ATC TAT GAA ATG TCG CTG TCG G
57445cre: CAG ACT AAT TCT ACC ACT TAC TGT TCA GAC CTC C
Gapdh fw: TGT CAA GCT CAT TTC CTG GTA TGA
Gapdh rev: CTT ACT CCT TGG AGG CCA TGT AG

### Determination of cytokine mRNA expression by qPCR

RNA was isolated from 2 × 10^6^ cultured CD4^+^ T cells using the QIAGEN RNeasy mini kit according to the manufacturer’s instruction. RNA was eluted in nuclease-free water, and its concentration was determined on a Nanodrop spectrophotometer (Thermo Fisher). From the total isolated RNA, 500 ng was reversely transcribed using the high-capacity cDNA reverse transcription kit (Thermo Fisher). Real-time qPCR was performed using a TaqMan master mix and the light cycler 96 (Roche) applying the following parameters: 2 min at 50°C, 10 min at 95°C, and then 40 cycles of denaturation at 95°C for 15 s and annealing–elongation at 60°C for 60 s. The Ct values for the individual samples (genes) were then normalized to β-actin gene expression as the internal control. To determine the Ct value change (ΔCt), the Ct value of the endogenous control gene was subtracted from the Ct value of each target gene. Then, the fold change was calculated to determine the relative expression of each gene. All the primer sets were purchased from Thermo Fisher: (Mm01168134_m), TNFα (Mm00443260_g1), IL-10 (Mm00439614_m1), and β-actin (Mm00607939_s1).

### Acid ceramidase enzymatic assay

CD4^+^ T cells (1 × 10^6^) were washed once with PBS and then resuspended in 0.1 M sodium acetate and 0.2% sodium taurocholate (adjusted to pH 5.2) before being subjected to four times freeze–thaw cycles (−80°C and 37°C). The mixture was centrifuged (13,000 rpm, 5 min, 4°C), and the supernatant was taken as the lysate. Sample lysate was incubated with 100 pmol NBD-C12-Ceramide (Cayman Chemical Company) in substrate buffer for 24 h at 37°C before spotted onto a thin layer chromatography (TLC) plate as described before (29). The plate was developed for 2 min under 600 nm using LI-COR Odyssey and quantified using ImageJ. Ac activity is demonstrated as % sphingosine of total lipid and calculated by division of the sphingosine signal to the total signal of sphingosine and ceramide.

### Intracellular sphingolipid quantification by HPLC-MS/MS

CD4^+^ T cells (1 × 10^6^) were washed twice with PBS, followed by resuspension in 500 µl methanol. Sphingolipids were extracted by the addition of 1 ml methanol/chloroform (1:1, v:v) as previously described (30). The extraction solution also contained the internal standards d_7_-sphingosine (d_7_-Sph; 0.25 pmol on column), C17:0 ceramide (C17:0 Cer, 2.5 pmol on column), and d_31_-C16:0 sphingomyelin (d_31_-C16:0 SM, 2.5 pmol on column) (all Avanti Polar Lipids, Alabaster, USA). Subsequent HPLC-MS/MS analysis was performed under already published conditions (31). Chromatographic separation of the lipid extract, 10 µl injected onto the column, was performed using a 1290 Infinity II HPLC (Agilent Technologies, Waldbronn, Germany) equipped with a Poroshell 120 EC-C8 column (3.0 × 150 mm, 2.7 µm; Agilent Technologies). MS/MS analyses were conducted using a 6495C triple–quadrupole mass spectrometer (Agilent Technologies) in positive electrospray ionization (ESI+) mode. The mass transitions of sphingolipid species recorded for multiple reaction monitoring (MRM), and the retention times can be found in the **Supplementary Table S2**. Cer and SM subspecies were quantified by external calibration, while Sph was directly quantified in relation to its internal standard d_7_-Sph. Peak areas were determined using MassHunter Quantitative Analysis Software (version 10.1, Agilent Technologies).

### Statistical analysis

Statistical analyses were conducted using GraphPad Prism 10 Software. To compare two groups under multiple parameters, two-way ANOVA Sidak’s multiple comparison was performed, whereas two-tailed unpaired t-test was used to compare two groups based on a single parameter. Data were presented as individual mouse/replicate together with means and standard deviation (SD) and were considered to be significantly different when p < 0.05 (*p<0.05, **p<0.01, ***p<0.001, ****p<0.0001).

## Results

### Induced knockout of Ac *in vivo* increases the ceramide content of CD4^+^ T cells

**Figure 1:**
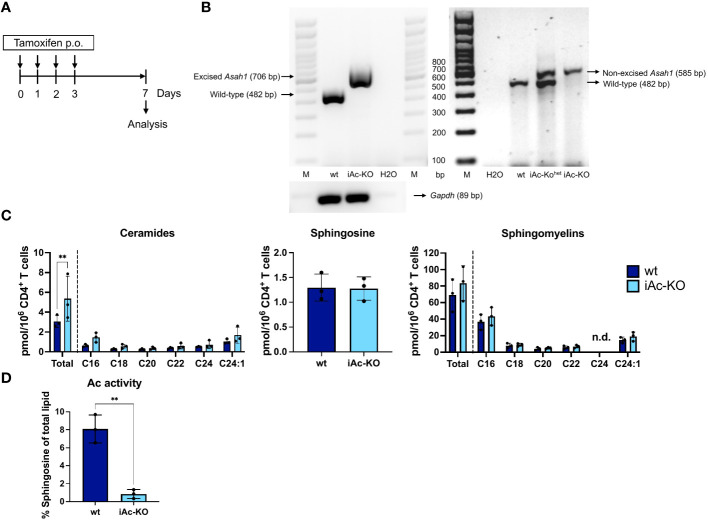
Tamoxifen administration to iAc-KO *in vivo* led to efficient gene recombination and subsequently to ceramide accumulation and reduced Ac activity. **(A)** *In vivo* experimental procedure to induce the knockout of Ac. Tamoxifen was administered for four consecutive days per oral. Mice were then sacrificed on day 7 for analysis. **(B)** DNAs of purified CD4^+^ T cells from iAc-KO and wt littermates on day 7 were isolated and subsequent PCR to validate the *Asah1* recombination (left panel). As comparison, genotyping results were shown to indicate non-excised band of *Asah1* (right panel). **(C)** The levels of ceramides, sphingosine, and sphingomyelins of purified CD4^+^ T cells of iAc-KO and iAc-wt on day 7 were measured by HPLC-MS/MS (n=3). **(D)** Lysates of day 7 CD4^+^ T cells of iAc-KO and Ac-wt were measured for their Ac enzymatic activity using TLC-based assay (n=3). All depicted graphs show data from individual mice together with means ± SD. Statistical analysis was performed by two-way ANOVA with Sidak’s multiple comparison test or unpaired t-test (**p<0.01).

In order to delete Ac expression *in vivo*, we applied tamoxifen to iAc-KO or wild-type (wt) littermate mice on four consecutive days and isolated CD4^+^ T cells for analysis after a further 3 days (**Figure 1A**). Genomic PCR detecting the wt *Asah1* gene, the floxed, but non-recombined, and the recombined *Asah1* allele showed that tamoxifen treatment had efficiently induced recombination (**Figure 1B**). *Asah1* recombination was associated with an increase in ceramide, while both sphingosine and sphingomyelin concentrations remained unaltered (**Figure 1C**). Reduced Ac activity further showed that *Asah1* recombination led to reduced expression of the enzyme (**Figure 1D**). Taken together, our data indicated that the *Asah1* gene and its expression were efficiently targeted in iAc-KO mice.

### The composition of the CD4^+^ and CD8^+^ T-cell compartment and T-cell activation and proliferation were largely unaltered after iAc-KO *in vivo*

**Figure 2:**
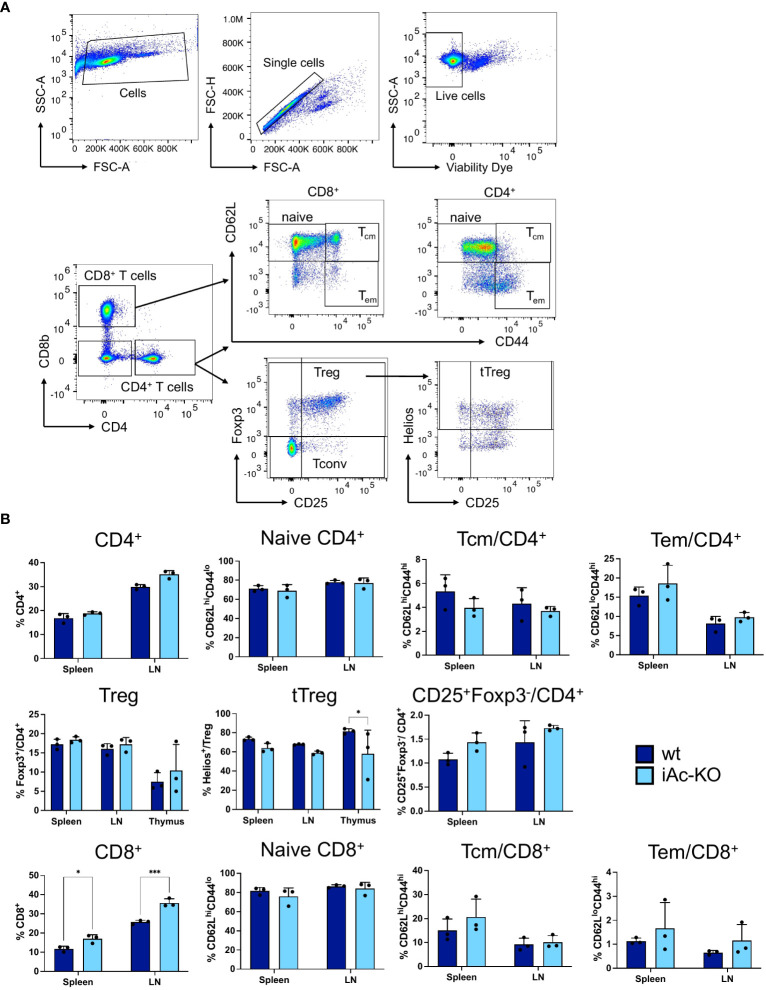
*In vivo* Ac deletion only had minor effects on subset composition of CD4^+^ and CD8^+^ T cells. **(A)** Representative dot plots showing the gating strategy for CD4^+^, CD8^+^ T cells, and their subsets. **(B)** Frequencies of CD4^+^ and CD8^+^ T cells together with their subsets in the spleen, lymph nodes, and thymus comparing iAc-KO to wt littermates on day 7 (n=3). Columns show each individual mice together with means ± SD. Two-way ANOVA followed by Sidak’s multiple comparison test was done for statistical analysis (*p<0.05, ***p<0.001).

To understand how induced Ac knockout affects the T-cell compartment, we determined absolute cell numbers and analyzed subpopulations by flow cytometry in the thymus, spleen, and lymph nodes. We observed lower frequencies of Helios^+^ cells among CD4-single positive Foxp3^+^ CD25^+^ thymic Treg (tTreg), a higher proportion of DN2 cells among double-negative thymocytes, and a higher proportion of CD8^+^ T cells among splenocytes and lymph node cells in iAc-KO versus wt mice (**Figures 2A, B**; **Supplementary Figure S1B**). Regarding absolute cell numbers, we observed lower naive CD4^+^ T cell and thymus-derived, i.e., Helios^+^, tTreg counts in the spleen of iAc-KO versus wt mice (**Supplementary Figure S2**).

To determine whether reduced frequencies and numbers of tTreg were due to Ac deficiency in Treg themselves or caused by the lack of Ac expression in other cells, we analyzed iAc-KO mice with tamoxifen-inducible Cre knocked into the Foxp3 locus (Foxp3-iAc-KO). In these mice, neither the proportion among CD4^+^ T cells nor absolute numbers of tTreg were changed (**Supplementary Figure S3A**).

**Figure 3:**
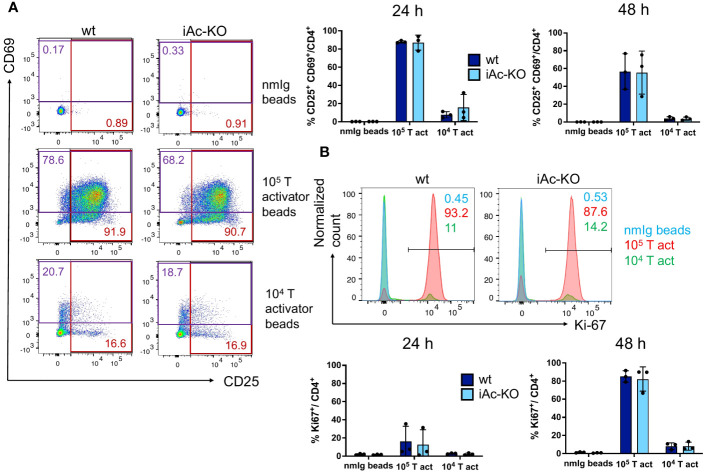
CD4^+^ T-cell activation and proliferation were not altered in after Ac knock-out. **(A)** CD4^+^ T cells of iAc-KO and iAc-wt day 7 were restimulated with 10^5^, 10^4^ T-activator beads and normal mouse Ig (nmIg) beads for 24 and 48 h. In the left panel, representative flow cytometric dot plots as example from three independent experiments depict the frequencies of single positive for activation markers CD25 and CD69 among CD4^+^ T cells upon different stimulation for 2 days. The frequency of CD4^+^ T cells that are double positive for CD25 and CD69 comparing iAc-KO to wt littermates after 24 and 48 h stimulation is shown in the right panel (n=3). **(B)** Proliferation of CD4^+^ T cells from the same culture was monitored. Representative histograms displayed proliferation (marker, Ki-67) of CD4^+^ T cells of iAc-KO and iAc-wt upon 2 days stimulation with T-activator beads and nmIg beads as negative control. The bar graphs are summarized data of the proliferating CD4^+^ T cells of iAc-KO and iAc-wt upon 1 and 2 days stimulation (n=3). Data are depicted from individual mice together with means ± SD. Statistical analysis was performed by two-way ANOVA with Sidak’s multiple comparison test.

Apart from the phenotype, we also tested the functionality of iAc-KO versus wt CD4^+^ T cells by *in vitro* stimulation with T-activator CD3/CD38 beads over 1 days and 2 days. Neither activation (% CD25^+^ CD69^+^) nor proliferation (% Ki-67^+^) differed between wt and iAc-KO CD4^+^ T cells (**Figure 3**). Apart from lower frequencies of tTreg among CD4^+^ T cells and a higher proportion of CD8^+^ T cells among lymph node cells and splenocytes, the T-cell compartment of iAc-KO mice appeared largely unaltered.

### CD4^+^ T cells from iAc-KO mice secreted more IFNγ and IL-5 than their wt counterparts

**Figure 4:**
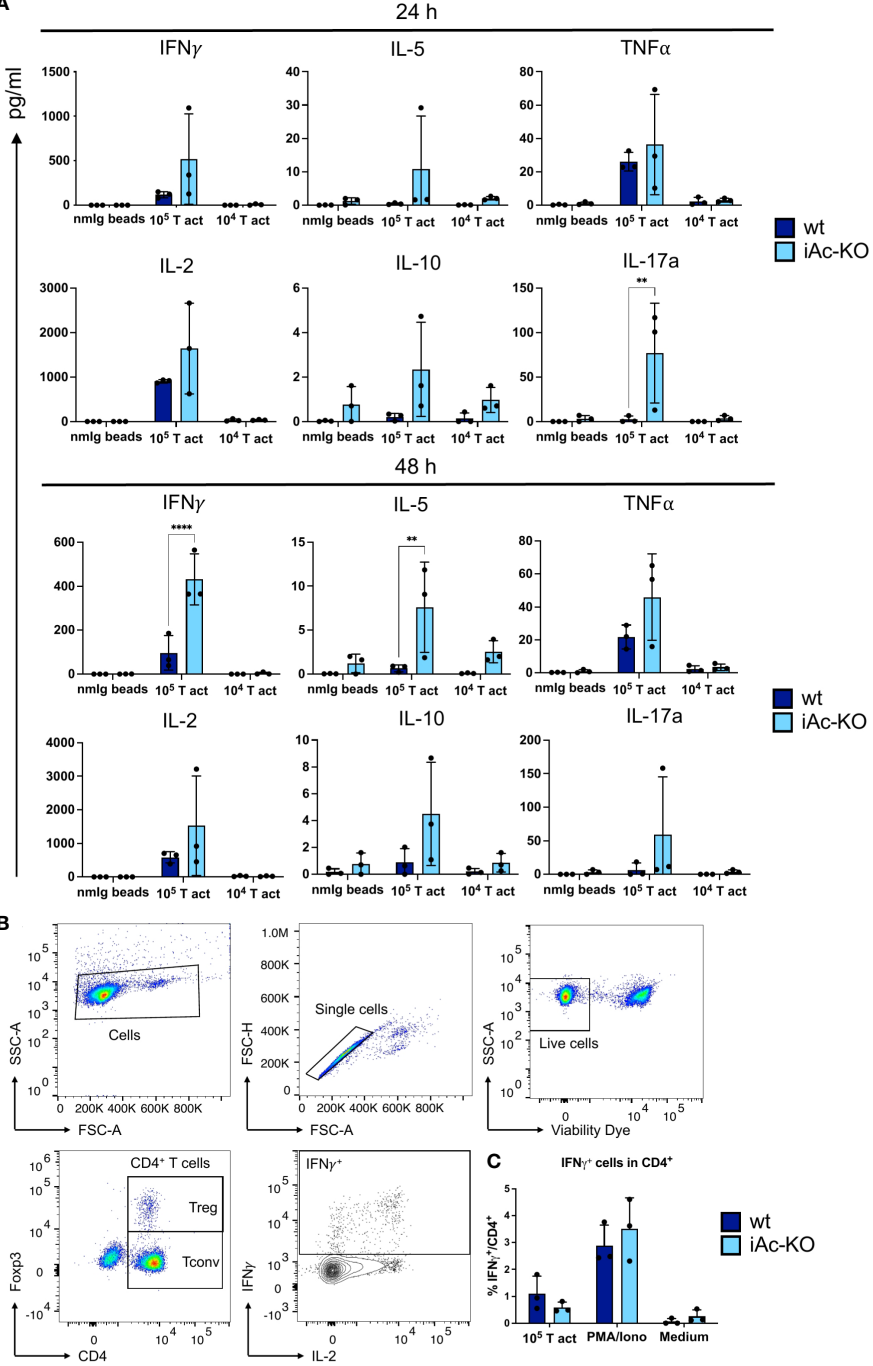
Ac-deficient CD4^+^ T cells secreted more upon optimal restimulation with 10e^5^ T-activator beads. **(A)** Supernatant from the 2 days stimulation culture of iAc-KO and iAc-wt CD4^+^ T cells were measured using bead-based assay and flow cytometry (n=3). **(B)** Flow cytometric gating strategy for IFNγ-producing cells among CD4^+^ T cells shown following intracellular cytokine staining. CD4^+^ T cells of iAc-KO and iAc-wt were stimulated with 10^5^ T-activator beads for 4 h in the presence of brefeldin A to block the cytokine secretion. PMA/Iono and medium were used as positive and negative control, respectively. **(C)** Frequency of IFNγ-producing cells among CD4^+^ T cells were then measured by flow cytometry comparing iAc-KO and wt CD4^+^ T cells (n=3). Data are compiled from three mice per genotype and shown together with means ± SD. Two-way ANOVA with Sidak’s multiple comparison test was done to test for statistical significance (**p<0.01, ****p<0.0001).

A key function of CD4^+^ T cells is to orchestrate immune responses through cytokine secretion. Therefore, we determined cytokine concentrations in the culture supernatants of mouse T-activator beads CD3/CD28-stimulated CD4^+^ T cells. After 24 h, IL-17a secretion was increased, while after 48 h, secretion of both IL-5 and IFNγ was higher for CD4^+^ T cells from iAc-KO compared to wt mice, with the amount of IFNγ secreted being approximately 10-fold higher than for the other two cytokines (**Figure 4A**). For the remaining cytokines, i.e., for IL-2, TNF, and IL-10, there was a trend towards higher secretion by iAc-KO versus wt CD4^+^ T cells. To determine whether differences in the proportion of cytokine-producing cells among CD4^+^ T cells or in the amount of cytokine secreted per cell accounted for higher concentrations in culture supernatants, we analyzed IFNγ-secreting cells by intracellular cytokine staining (**Figures 4B, C**). As the intracellular staining for IFNγ did not reveal any differences between iAc-KO and wt CD4^+^ T cells, the increased amounts of IFNγ that we had detected in culture supernatants were not due to differences in expression but in secretion of IFNγ.

### iAc-KO *in vitro* followed by CD28-superagonist-driven expansion allowed studying cell-intrinsic effects in CD4^+^ T cells

**Figure 5:**
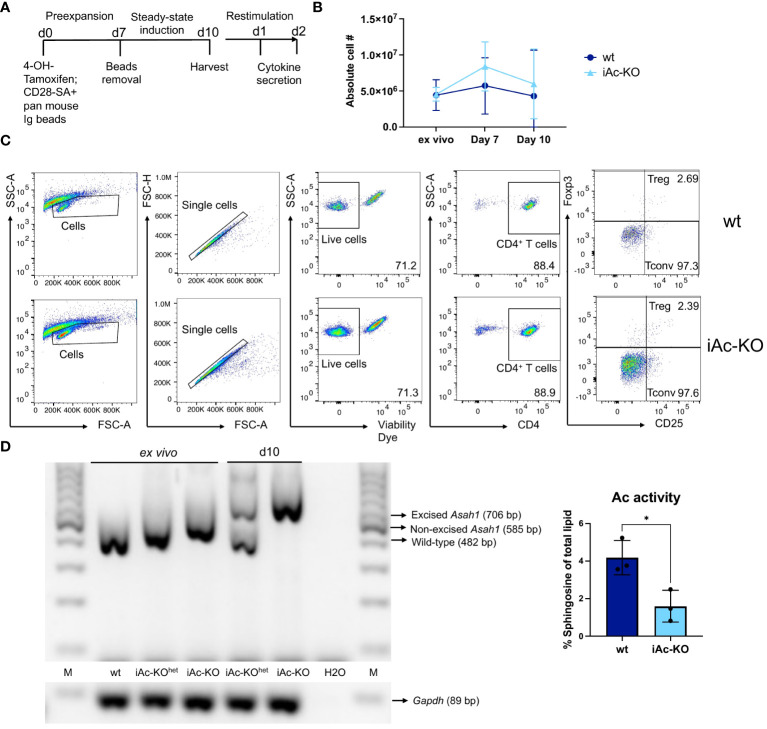
The recombination of Ac gene (*Asah1*) could be induced *in vitro* and CD4^+^ T cells subsequently maintained in *in vitro* culture. **(A)** *In vitro* experimental setup for CD4^+^ T-cell pre-expansion and restimulation culture. Cells from the spleen and lymph nodes were treated with 4-OH-Tm for 1 h, which is followed by CD4^+^ purification. CD4^+^ T cells were then cultured in the presence of CD28-Superagonist (CD28-SA) and pan mouse Ig beads for 7 days. On day 7, beads were removed, and the cells were washed before seeded in only medium for another 3 days to induce steady state. Cells were harvested on day 10 and restimulated for 24 h and 48 h with T-activator beads and nmIg beads. **(B)** CD4^+^ T cells yield of iAc-KO and iAc-wt on day 0, 7, and 10 (n=3). **(C)** Representative gating strategy showing Treg and Tconv of iAc-KO and its wt littermates after 10 days of culture. **(D)** DNA of *ex vivo* spleen/LN cells treated with 4-OH-tamoxifen and day 10 CD4^+^ T cells of iAc-KO (and wt littermates) was isolated and used for PCR to validate *Asah1* recombination after 10 days of culture (left panel). Lysates of day 10 CD4^+^ T cells from iAc-KO and iAc-wt were measured for its Ac activity (right panel). Data are shown as converted sphingosine of total lipid (n=3). All depicted graphs show data from individual mice together with means ± SD. Statistical analysis was performed by two-way ANOVA with Sidak’s multiple comparison test or unpaired t-test (*p<0.05).

While Ac deletion was very efficient in iAc-KO mice (**Figure 1**), studying CD4^+^ T cells from iAc-KO mice did not reveal whether increased cytokine secretion by CD4^+^ T cells was due to Ac deletion in CD4^+^ T cells themselves or in other cells. To analyze this, we established an *in vitro* culture system where we first induced recombination in total spleen/lymph node cells, then isolated CD4^+^ T cells followed by a first culture in the presence of a superagonistic anti-CD28 mAb (CD28-SA) and cross-linking Pan Mouse Ig Dynabeads™ (**Figure 5A**) (32). On day 7, Dynabeads were removed, and cells were seeded again in mouse medium without further supplements to induce a resting state. By day 10, absolute cell numbers were similar to those that the cultures had been initiated with (**Figure 5B**), Treg frequencies were between 2% and 3% for both genotypes, and the degree of CD25 expression among Foxp3^-^ CD4^+^ T cells indicated a resting or near-resting state of conventional T cells (**Figure 5C**). Similar to the *in vivo* setting, *Asah1* gene recombination was very efficient *in vitro*, and by day 10, Ac activity was clearly reduced (**Figure 5D**), indicating that also the *in vitro* culture system allowed us to efficiently generate Ac-deficient CD4^+^ T cells for further analysis. Regarding direct detection of Ac protein expression by Western blotting, this has been successful for mouse airway epithelia (33), L929 mouse fibroblasts (31), and mouse macrophages (25). Using the same polyclonal anti-Ac antibody, we, however, did not detect Ac protein in wt mouse CD4^+^ or CD8^+^ T cells (**Supplementary Figure S6**). This was in contrast to the mouse heart, i.e., the organ with the highest expression of Ac in humans according to The Human Protein Atlas (**Supplementary Figure S6**). Therefore, measuring Ac activity (**Figures 1D**, **5D**) was the closest we could get to determine the impact of induced gene deletion on Ac protein expression.

### Knockout of the Ac in isolated CD4^+^ T cells is sufficient to increase IFNγ secretion

**Figure 6:**
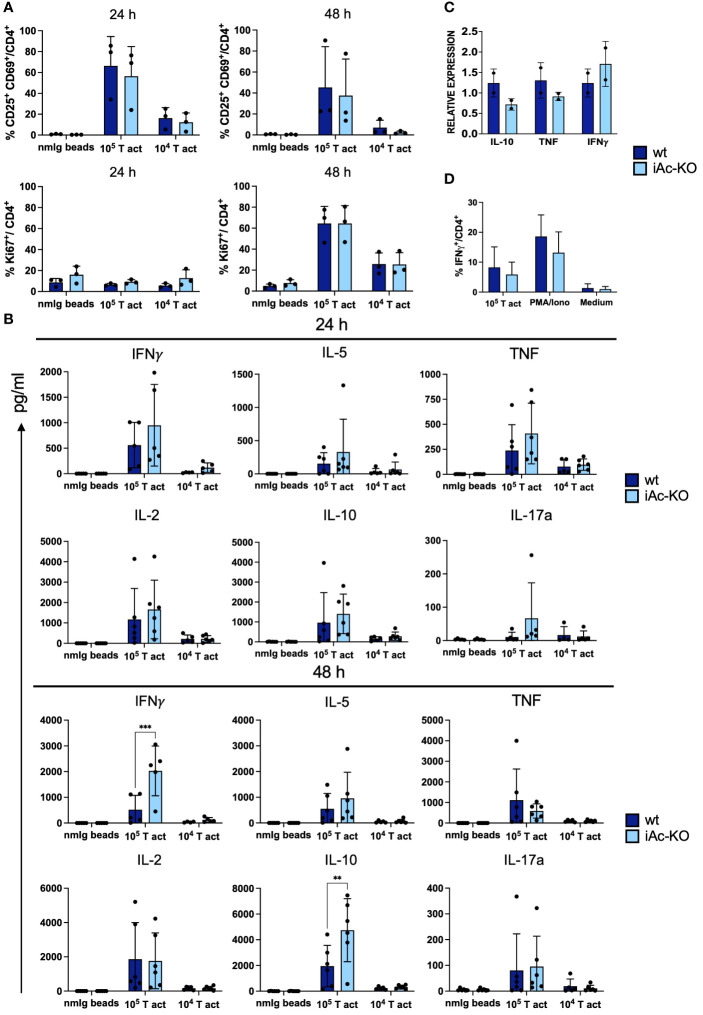
Increase in IFNγ secretion by Ac-deficient CD4^+^ T cells was evident upon restimulation following *in vitro* pre-expansion. **(A)** Frequencies of double-positive CD25 and CD69 among CD4^+^ T cells from iAc-KO and iAc-wt representing activation (left panel) and proliferation using Ki-67 as marker (right panel) (n=3). On day 10, CD4^+^ T cells were restimulated with different concentration of T-activator beads. As negative control, nmIg beads were employed. **(B)** Cytokines were measured using supernatant originated from restimulation culture comparing Ac-deficient CD4^+^ T cells to wt littermates (n=4–6). **(C)** Cytokine mRNA expression profiles of wt and iAc-KO CD4^+^ T cells after restimulation with T-activator beads for 4 h at a 1:1 bead to cell ratio (culture triplicates; experiment was repeated with similar result) **(D)** Intracellular cytokine staining was performed with CD4^+^ T cells of iAc-KO and iAc-wt on day 10 of culture using different stimulation (T-activator beads, PMA/Iono, and medium) for 4 h in the presence of brefeldin **(A)** The frequency of IFNγ-producing cells among CD4^+^ T cells upon different stimulation is summarized in the graph (n=2). All depicted data show means ± SD. Two-way ANOVA with Sidak’s multiple comparison test was done to test for statistical significance (**p<0.01, ****p<0.0001).

We stimulated *in vitro* generated Ac-deficient CD4^+^ T cells under the same culture conditions as CD4^+^ T cells isolated from iAc-KO mice observing that, again, neither activation nor proliferation of CD4^+^ T cells from both genotypes differed (**Figure 6A**). Regarding cytokine secretion, *in vitro*-generated Ac-deficient CD4^+^ T cells secreted more IFNγ and IL-10 compared to their wt counterparts (**Figure 6B**). mRNA expression (**Figure 6C**; **Supplementary Figure S8**) and intracellular cytokine staining (**Figure 6D**) confirmed that IFNγ expression was also not altered after Ac deletion *in vitro*. *In vitro* deletion of Ac expression in isolated CD4^+^ T cells, thus, revealed that Ac expression in CD4^+^ T cells regulated both IFNγ and IL-10 secretion.

### Ac expression in B cells is required to maintain mature B cell and plasma blast cell numbers

**Figure 7:**
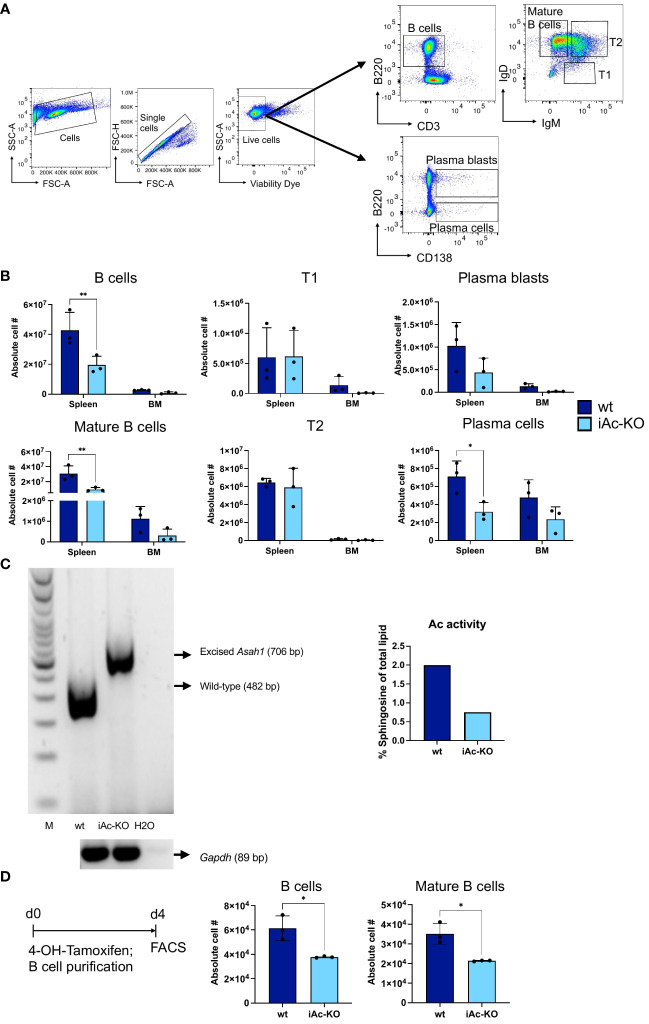
Global Ac ablation resulted in B-cell number reduction *in vivo* and *in vitro*. **(A)** Representative flow cytometric gating strategy for B cells and its subsets on day 7 after tamoxifen *in vivo* treatment. **(B)** Absolute cell number of B cells and their subsets comparing iAc-KO and iAc-wt in spleen and bone marrow *ex vivo* on day 7 (n=3). **(C)** DNA of iAc-KO and iAc-wt B cells after 4 days of culture were employed to confirm the recombination via genomic PCR (left panel). Ac enzymatic activity was also measured from the isolated B cells on day 4 and shown as representative (right panel). **(D)** *In vitro* B-cell culture setup (left panel). Cells from the spleen were treated with 4-OH-tamoxifen for 1 h and subsequently purified for B cells. B cells were cultured for 4 days before analyzed. Representative absolute cell number of B cells and mature B cells was determined on day 4 of culture (right panel) (n=3 replicates). Experiment was repeated and showed same results. Data are depicted in means ± SD. Two-way ANOVA with Sidak’s multiple comparison test or unpaired t-test were performed for statistics (*p<0.05, **p<0.01).

As our phenotypic analysis of iAc-KO mice (**Figure 1A**) also included the B-cell compartment, we detected that Ac deficiency led to a reduction both in absolute numbers and in the proportion of B cells among splenocytes in these animals compared to wt mice (**Figures 7A, B**; **Supplementary Figure S4**). Within the B-cell lineage, it was mature and plasma blasts and cells that were reduced. In contrast, frequencies among B cells and absolute cell numbers of immature T1 and T2 cells were either not altered or increased (**Figure 7B**; **Supplementary Figure S4**). Similar to the *in vitro* cell culture system set up for T cells, we also induced Ac deficiency *ex vivo* followed by isolation and culture of separated B cells (**Figure 7C**). Due to the notoriously poor survival of B cells *in vitro*, we cultured the B cells only for 4 days. Our preliminary data indicated that this period was sufficient for *Asah1* gene recombination and reduction of Ac activity in B cells (**Figure 7C**). *In vitro* deletion of the Ac in isolated B cells was sufficient to reduce B-cell recovery by approximately 50% (**Figure 7D**). Staining the cultured B cells with Annexin V and propidium iodide further suggested that iAc-KO B cells were more prone to undergo apoptotic cell death than their wt counterparts (**Supplementary Figure S7**). Ac expression in mature and further differentiated B cells, thus, was required to maintain B-cell numbers.

## Discussion

Here, we describe phenotypic changes in cells of adaptive immunity after induced deficiency for Ac. Our main findings were that Ac deletion increased cytokine, predominantly IFNγ, secretion by CD4^+^ T cells, and led to a loss of mature B cells.

A recent study using Ac-floxed mice together with a T-cell-specific Cre (Ac-floxed-CD4Cre) revealed that Ac deletion in T cells slowed melanoma progression (27). This was associated with increased expression of IFNγ and granzyme B by CD4^+^ and CD8^+^ T cells of tumor-bearing Ac-CD4Cre mice compared to wt littermates. Moreover, the authors observed enhanced TCR signaling, i.e., stronger phosphorylation of ZAP-70 and PLCγ1, in and activation of T cells of Ac-deficient compared to wt T cells. This is in contrast to our observations, as we did not detect differences in short-term activation and proliferation of CD4^+^ T cells from iAc-KO versus wt mice (**Figures 3**, **6**). Despite this, iAc-KO CD4^+^ T cells also secreted more cytokines, particularly IFNγ, after CD3/CD28 costimulation compared to CD4^+^ T cells of wt littermates (**Figures 4A**, **6B**).

To exclude effects in trans, i.e., due to Ac deficiency in non-CD4^+^ T cells of iAc-KO mice, we tried to use tamoxifen-inducible CD4-Cre mice [B6(129X1)-Tg(Cd4-cre/ERT2)11Gnri/J] available from Jackson Laboratory. However, in CD4^+^ T cells of these mice, we did not observe recombination of the floxed *Asah1* gene (**Supplementary Figure S5**). This was, of course, in contrast to Foxp3-iAc-KO mice showing efficient recombination (**Supplementary Figure S3B**).

As the tamoxifen-inducible CD4-Cre mouse line tested by us was not useful, we set up an *in vitro* assay system to study the impact of Ac deletion in isolated CD4^+^ T cells. These experiments comprising Ac deletion *in vitro* revealed that only secretion of IFNγ and IL-10 were controlled by Ac expression in CD4^+^ T cells. As for CD4^+^ T cells from iAc-KO mice, *in vitro*-generated Ac-deficient CD4^+^ T cells were not easier to activate than their wt counterparts (**Figure 6**).

A key requisite of the *in vitro* system was that it allowed for downregulation of Ac protein expression prior to interrogating T-cell function (**Figure 5**). The same was true after induced KO of Ac expression *in vivo* (**Figure 1**) where we also observed an increase in overall ceramide content in CD4^+^ T cells of iAc-KO versus wt mice, while neither the amounts of sphingomyelins nor sphingosine retrieved per cell was changed (**Figure 1**). While changes in sphingomyelins are *per se* much more difficult to observe than changes in ceramides due to the much higher abundance of sphingomyelins than ceramides in cells, we currently do not know how sphingosine levels were maintained in Ac-deficient CD4^+^ T cells. Generally, the concentration of sphingosine is very tightly regulated within cells due to the toxicity associated with higher concentrations of sphingosine (34). Therefore, changes in sphingosine generation in lysosomes cannot be expected to lead to a change in overall sphingosine content of cells. Still, we assume that the changes in ceramide content pinpoint to the biochemical mechanism underlying the changes in cytokine secretion that we observed in iAc-KO versus wt mice and that sphingosine plays a crucial role here. As the Ac is a lysosomal enzyme, the lack of Ac activity leads to an accumulation of ceramide in lysosomes and, thus, to reduced supply of sphingosine into the salvage pathway (3). Through the salvage pathway, sphingosine is transported back to the ER where it is used to build ceramide. As the ER also supplies the Golgi apparatus with sphingolipids, we assume that reduced generation of sphingosine in the lysosome might lead to changes in the content of ceramide and possibly other sphingolipid species in the Golgi apparatus. Very recently, the proteins NPC1 and LIMP-2/SCARB2, which had previously only been recognized as cholesterol transporters, have been identified to mediate sphingosine transport from the lysosome to other cellular compartments (35).

In double mutant mice with deficiencies for *Asah1* and *Smpd1* (encoding for acid sphingomyelinase, Asm), ceramide accumulation and the pathophysiological stigmata of Farber disease are largely absent compared to single knockout mice for *Asah1* (36). Comparing these two mutants with wt littermates will, in the future, help to clarify whether the phenotypes that we observed were due to ceramide accumulation or the lack of sphingosine generation in lysosomes in the absence of Ac expression.

We used intracellular cytokine staining to show that Ac-deficient CD4^+^ T cells did not differ from their wt counterparts when it comes to cytokine synthesis in the ER (**Figure 4C**). Here, T cells from Ac-deficient mice rendered very similar results to those of Asm-deficient mice in that cytokine production in the ER, as detected by intracellular cytokine staining, was not altered compared to wt mice (37). Therefore, both in Ac- and in Asm-deficient mice, changes in the Golgi apparatus must account for the observed phenotypes regarding cytokine secretion. Based on the published findings discussed in the previous paragraph, we assume that it is the changes to sphingolipid content in the Golgi apparatus that lead to increased cytokine secretion by Ac-deficient cells.

Among the cytokines analyzed by us, IFNγ stuck out as its secretion by CD4^+^ T cells was elevated upon aCD3/aCD28 costimulation both after *in vivo* deletion of Ac expression and after *in vitro* Ac knockout and CD4^+^ T-cell isolation (**Figures 4A**, **6B**). Apart from IFNγ, IL-10 secretion was also elevated after Ac-KO *in vitro* (**Figure 6B**). In contrast, we observed elevated TNF secretion only by CD4^+^ T cells isolated from iAc-KO mice (**Figure 4A**), but not after Ac-deletion and CD4^+^ T-cell isolation *in vitro* (**Figure 6B**). Published data show that both IFNγ and IL-10 form particularly small spots when stained and analyzed by confocal microscopy and remain in close proximity to the Golgi apparatus until their release into the cleft of the immunological synapse (38, 39). In contrast, confocal images of TNF depict less condensed cumulation and a loss of association with the Golgi apparatus leading to “multidirectional” secretion of the cytokine (38, 39). These differences might provide the key to explain why IFNγ and IL-10 secretion are more strongly affected by Ac deficiency than TNF secretion and secretion of the other cytokines that we analyzed. Future experiments will, thus, focus on the subcellular localization of IFNγ, IL-10, TNF, and other cytokines and the mechanism of secretion in the absence of Ac expression. To increase the frequency of cytokine secreting cells, *in vitro* polarized Th1 and Th2 cells could be studied. We assume that cytokine secretion will also be increased by Ac-deficient Th1 and Th2 cells after *in vitro* differentiation as was the case for the naturally induced cytokine-secreting CD4^+^ T cells analyzed in this study.

Increased IFNγ secretion by CD4^+^ T cells might also contribute to the pathogenesis of FD by activating macrophages, thus increasing phagocytosis of sphingolipid-containing material from cells dying due to sphingolipid overload. Currently, it is, however, unknown whether CD4^+^ T cells of FD patients show increased IFNγ secretion.

Regarding the control of chronic viral infections such as persistent measles virus infection of the brain, pharmacologically blocking Ac activity (40, 41) or genetic approaches to knock out Ac expression in T cells might enhance viral clearance through enhanced IFNγ secretion. An also increased secretion of IL-10 might be beneficial in such a scenario, as it can be expected to prevent overshooting immune responses, which is reflected by the fact that thoroughly differentiated Th1 cells are well known to secrete IL-10, limiting collateral damage during pro-inflammatory immune responses (42, 43). Apart from increasing anti-viral immunity, pharmacological inhibition of the Ac with Ceranib-2 has been shown to directly inhibit measles virus growth (40). In addition, future experiments should address the role of Ac expression in cytotoxic CD8^+^ T-cell function and NK cells—both key mediators of anti-viral immunity. Based on published data using Asm-deficient mice (37) and our own findings on Ac-deficient CD4^+^ T cells, we assume that not only IFNγ secretion but also killing by CD8^+^ T cells will be increased in the absence of Ac expression.

While the composition of the T-cell compartment was largely unaltered in iAc-KO compared to wild-type mice, B-cell numbers were reduced (**Figure 7**). Apart from mature follicular B cells, also plasma blasts and cells, but not transitional B cells, were negatively affected by Ac deletion. Again, we set up a suitable *in vitro* cell culture system to study the effects on B cells after induction of Ac deletion *in vitro* and B-cell isolation revealing that Ac expression in B cells themselves was required to maintain their numbers. Currently, it is unclear why B cells are more sensitive to Ac deletion than T cells. A possible explanation could be that, unlike T cells, B cells do not express caveolin, which means that they depend more heavily on lysosomal activity than T cells to maintain the integrity of the cell membrane (44). Whether the lysosomal dysfunction observed in Ac-deficient cells (45) extends to an ineptitude to properly repair cell membrane damage is, however, unknown. Our own observation that liposomes preferentially enhance B- versus T-cell survival *in vitro* (46) might support the notion that B cells are in higher demand for lipids than T cells generated either by the cells themselves or acquired from their environment.

Taken together, the results of this study highlight a key role of the Ac in cytokine secretion by CD4^+^ T cells and possibly other lymphocyte subsets and an important contribution to B-cell homeostasis. In the future, we will further interrogate and seek to therapeutically exploit the role of the Ac in chronic viral infection models.

## Data availability statement

The original contributions presented in the study are included in the article/**Supplementary Material**. Further inquiries can be directed to the corresponding author.

## Ethics statement

The animal study was approved by the Regierung von Unterfranken as the responsible regulatory body (permission number: RUF-55.2.2-2532-2-1509-24). The study was conducted in accordance with the local legislation and institutional requirements.

## Author contributions

PM: Data curation, Formal analysis, Investigation, Methodology, Validation, Writing – original draft. CH: Methodology, Writing – review & editing. RZ: Formal analysis, Methodology, Writing – review & editing. SL: Formal analysis, Methodology, Writing – review & editing. JS: Formal analysis, Methodology, Writing – review & editing. DW: Formal analysis, Methodology, Writing – review & editing. FS: Formal analysis, Methodology, Writing – review & editing. BK: Conceptualization, Writing – review & editing. NB: Conceptualization, Funding acquisition, Resources, Supervision, Writing – original draft, Formal analysis.
