# Supplementary material for: Acid ceramidase expression reduces IFNγ secretion by mouse CD4+ T cells and is crucial for maintaining B-cell numbers in mice
Source: Front Immunol. 2024 Jun 11;15:1309846. doi: 10.3389/fimmu.2024.1309846 (PMC11196608; doi:10.3389/fimmu.2024.1309846)
Supplement: Supplementary file 1 [file DataSheet_1.pdf]

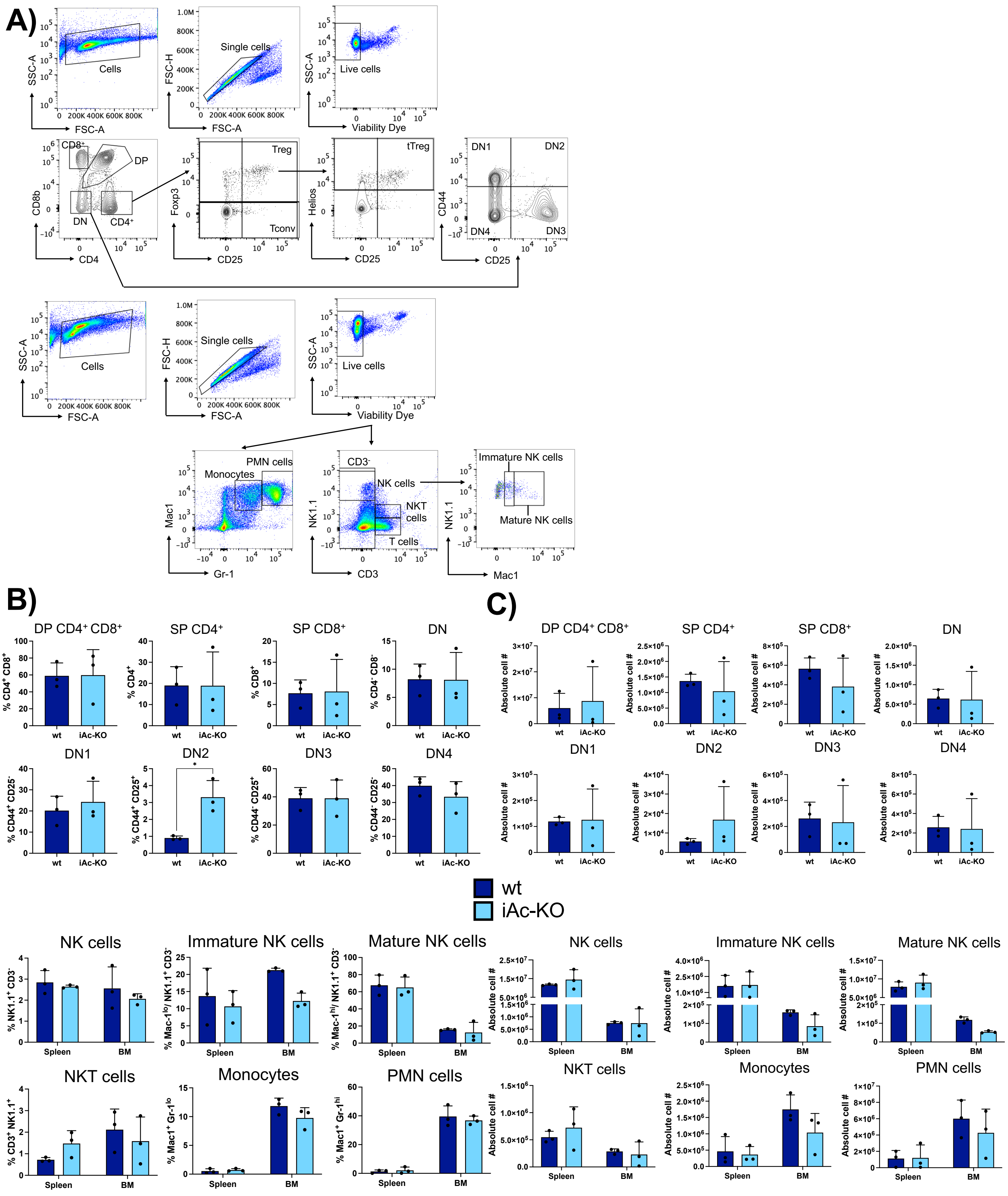

Suppl. Fig. 1. *In vivo* day 7 immunophenotyping for innate and adaptive immune cells following Ac ablation. **(A)** Gating strategy for T cells precursors in thymus (top) and NK cells, monocytes as well as polymorphonuclear (PMN) cells (below) **(B)** Frequencies and **(C)** absolute cell number of T cells precursors in thymus (top) and NK cells, monocytes and PMN cell in bone marrow and spleen of iAc-KO and wild-type mice on day 7 after tamoxifen administration (n=3). Each data point represents one individual mice and showed together with means ± SD. Statistical significance was conducted using two-way ANOVA and Sidak's multiple comparison test (*p<0.05).

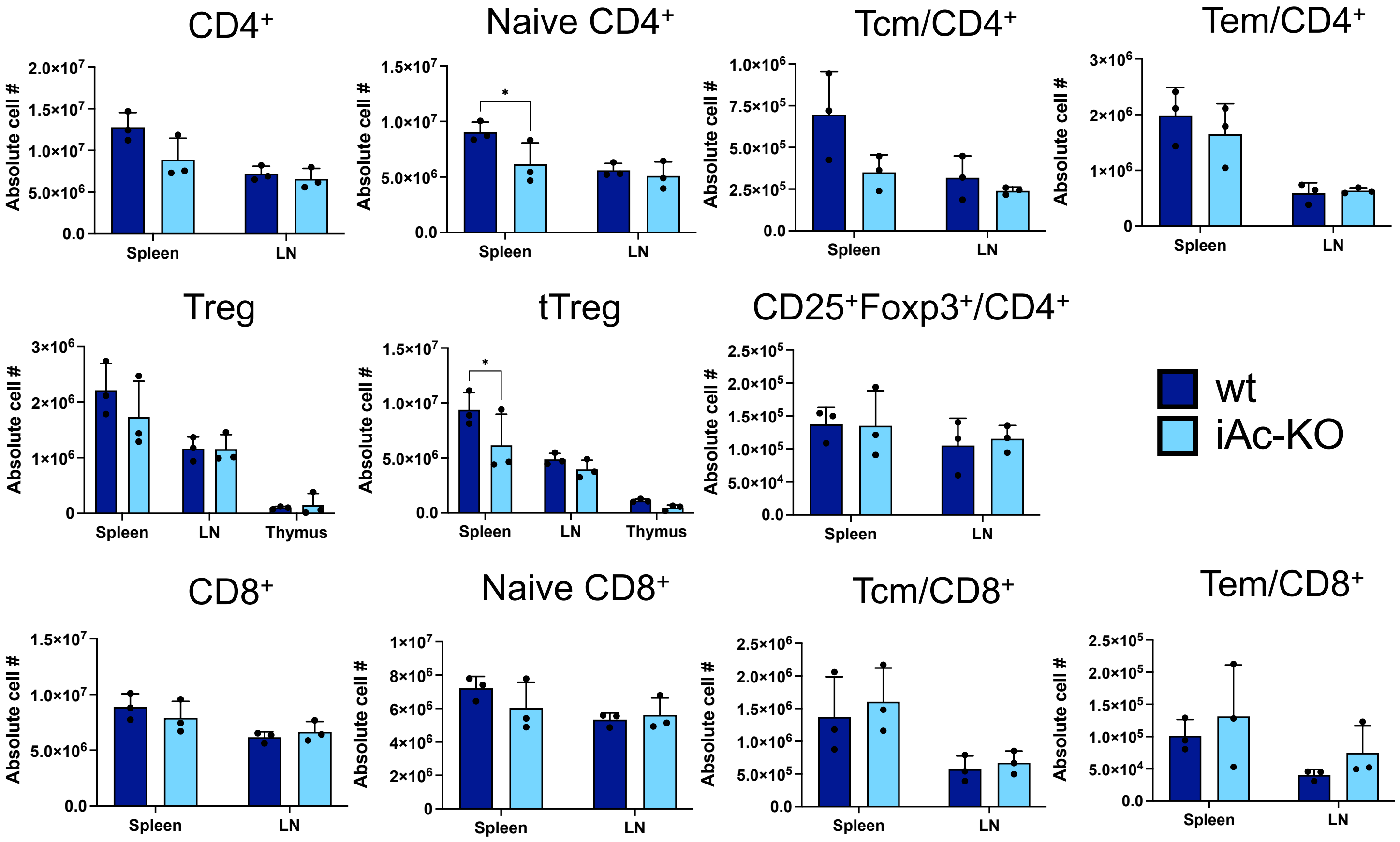

Suppl. Fig. 2. Absolute cell number of CD4+ and CD8+ T cells subsets of iAc-KO and wild-type mice from *in vivo* day 7 analysis (n=3). Data represents each individual mice with means ± SD. Statistical analysis was done using two-way ANOVA and Sidak's multiple comparison test (*p<0.05).

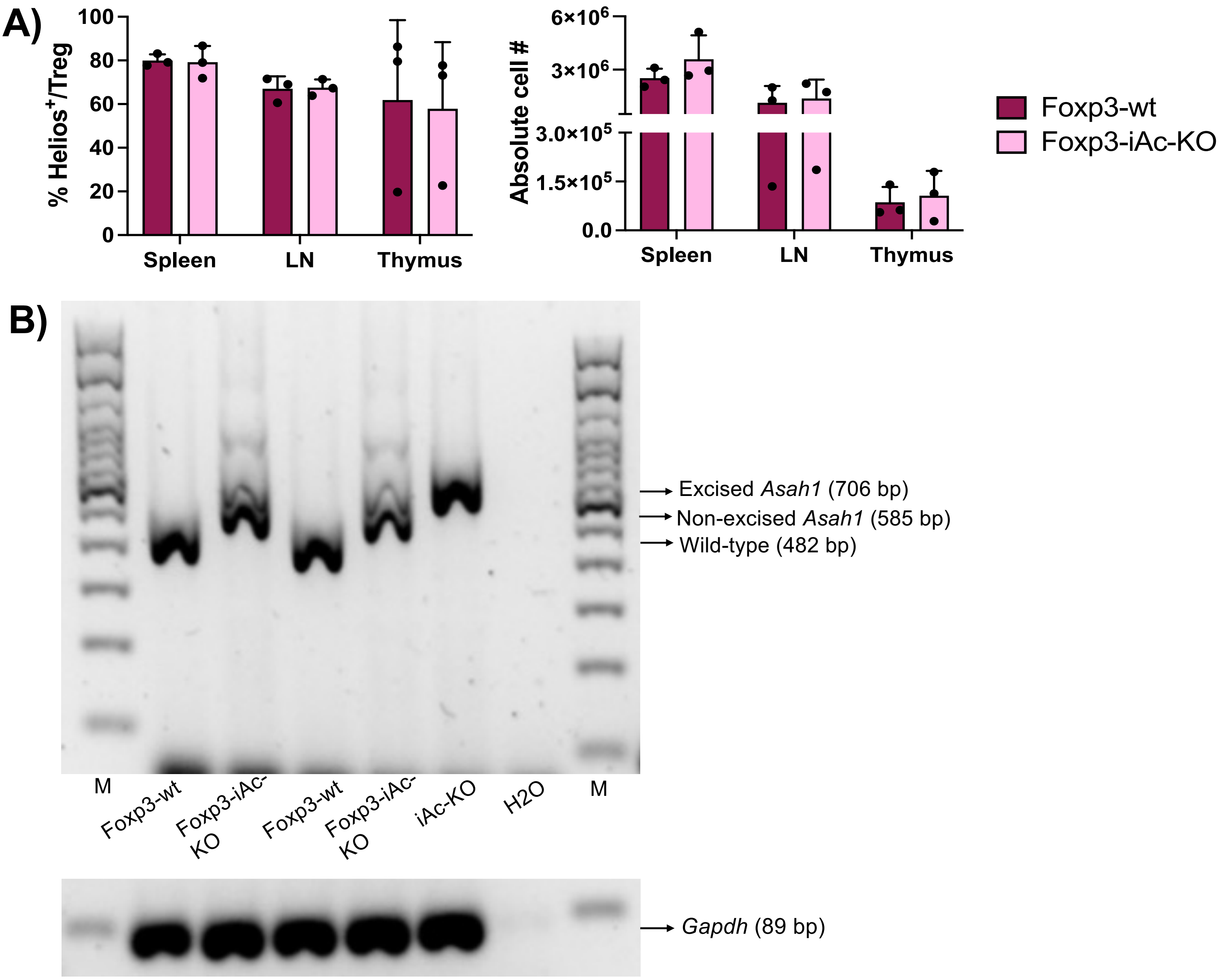

Suppl. Fig. 3. (A) Frequency and absolute cell number of tTreg in different lymphoid tissues of Foxp3-iAc-KO and Foxp3-wt mice administered with tamoxifen (n=3). Each individual mice is displayed with means ± SD and tested for statistical significance using two-way ANOVA followed by Sidak's multiple comparison test. **(B)** Recombination of *Asah1* of Foxp3-iAc-KO and its wild-type mice upon *in vivo* tamoxifen feeding.

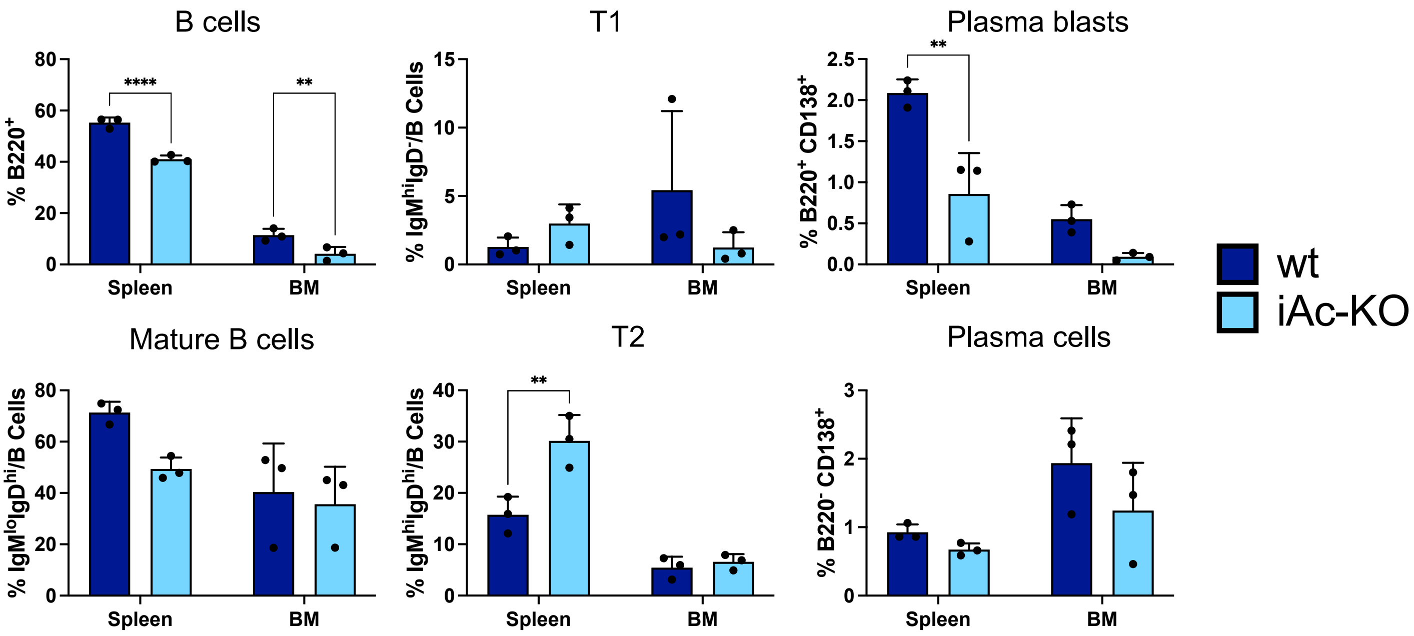

Suppl. Fig. 4. Frequencies B cells and its subsets of iAc-KO and control mice analyzed on day 7 after being fed with tamoxifen (n=3). Columns show each individual mice together with means ± SD. Two-way ANOVA followed by Sidak's multiple comparison test was done for statistical analysis (**p<0.01, ****p<0.0001).

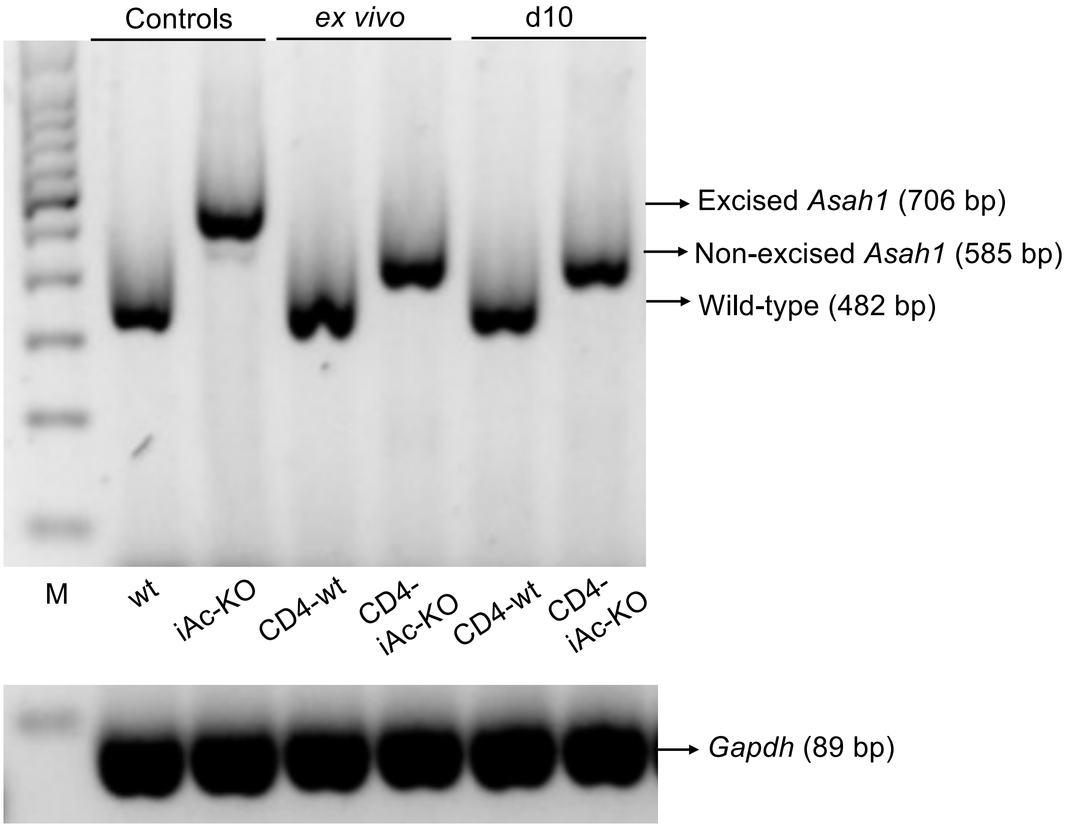

Suppl. Fig. 5. Validation of recombination using CD4-ERCre mouse line. CD4+ T cells were isolated and pre-expanded with CD28-Superagonist and pan mouse IgG beads for 7 days and induced for steady state for another 3 days. On day 10, DNA was isolated and used for PCR to confirm the recombination of *Asah1*.

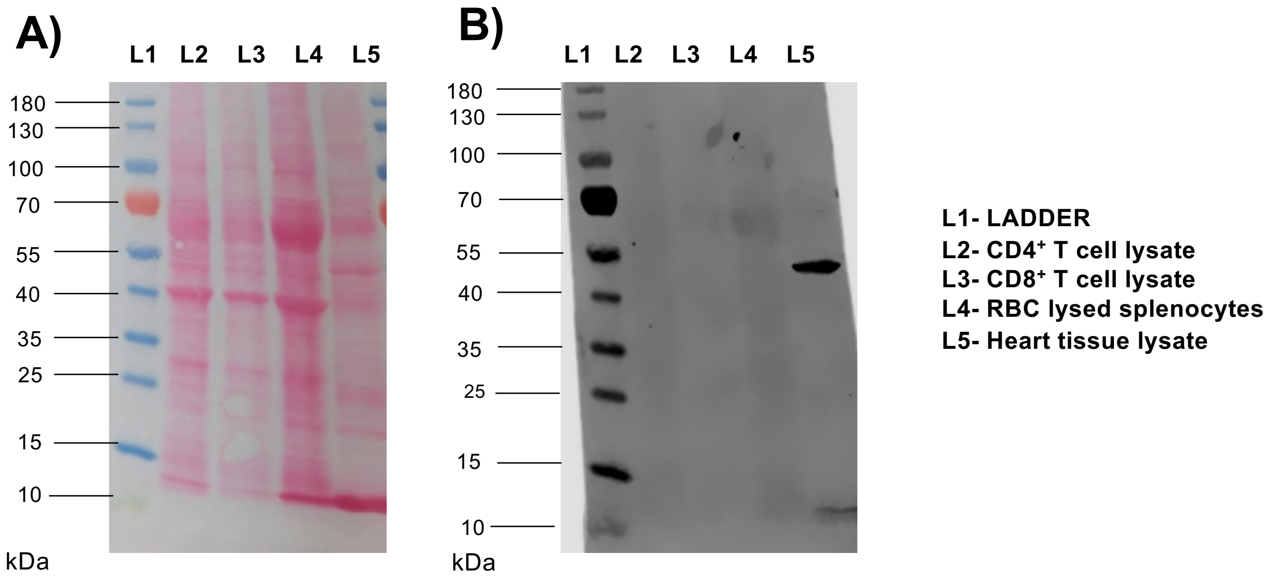

Suppl. Fig. 6. Western blot analysis to detect acid ceramidase protein expression in different cell types and tissue. 6 x 10^6 purified primary mouse $CD4^+$ or $CD8^+$ T cells, RBC-lysed total splenocytes and heart tissue were used to prepare the lysate by lysing the cells or tissue in 50 μl of RIPA buffer (Sigma-Aldrich, R0278) with 1x protease inhibitor cocktail (Sigma-Aldrich) and protein concentration was determined by using Bradford Assay kit (Thermofisher). **(A)** Protein loading was confirmed by Ponceau staining of the membrane. **(B)** Blocking was performed with 5% skimmed milk in PBS for 1 h at room temperature. Rabbit anti-Ac antibody (ProSci, 4741) was added as 1:500 dilution and incubated overnight at 4°C. HRP-conjugated anti-rabbit IgG antibody (Beckman Coulter) was used as 1:2000 dilution and incubated for 1 h at room temperature. Ac protein was only detected in heart tissue lysate (L5), but not in $CD4^+$ (L2) or $CD8^+$ T cells (L3) or RBC-lysed splenocytes (L4). The experiment was repeated twice with similar result.

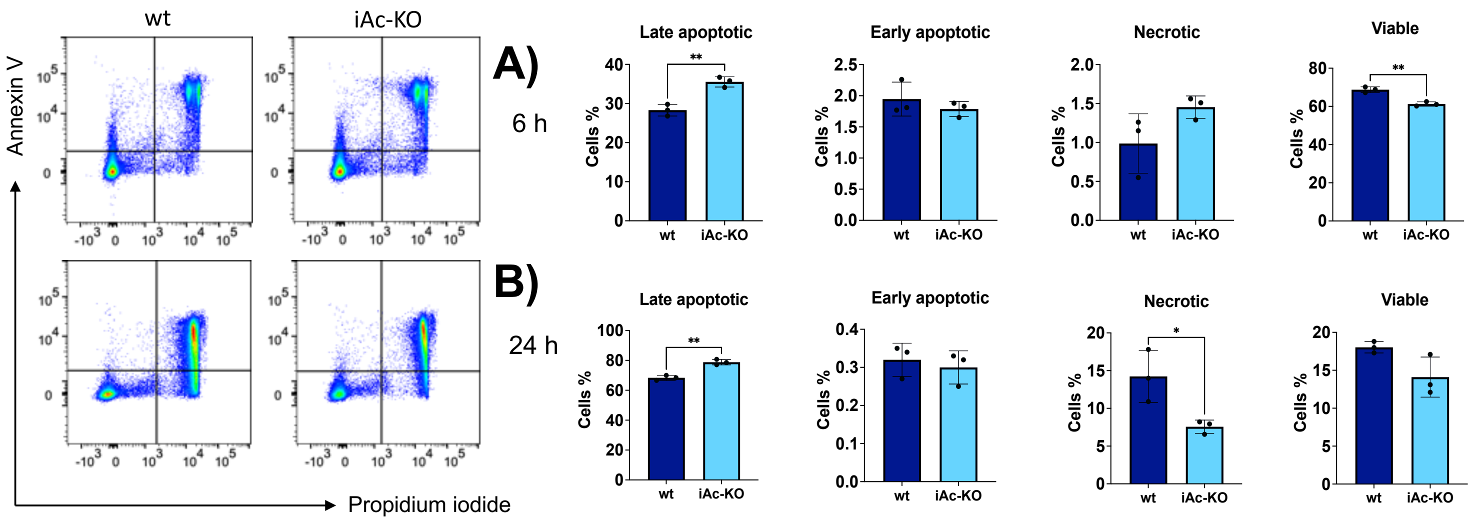

Suppl. Fig. 7. After induction of Ac deletion *in vitro,* B cells were purified and cultured for **(A)** 6 or **(B)** 24 h. At both time points the cells were stained using the FITC Annexin V Apoptosis Detection Kit with PI (Biolegend) following the manufacturer's instructions. iAc-KO B cells contained significantly higher proportions of late apoptotic cells (Annexin V^{+} PI^{+}) than wt B cells both after 6 and 24 h. No differences were observed for early apoptotic cells (Annexin V^{+} PI^{-}). The proportion of necrotic cells (Annexin V^{-}/PI^{+}) was higher among wt than iAc-KO B cells after 24 h (B), but not after 6 h (A). The proportion of viable cells (Annexin V^{-}/ PI^{-}) was lower among iAc-KO than wt B cells after 6 h of culture (A). Means ± SD of triplicate cultures are shown. An unpaired student's t-test was used to statistically analyze the data (*p<0.05, **p<0.01).

A)

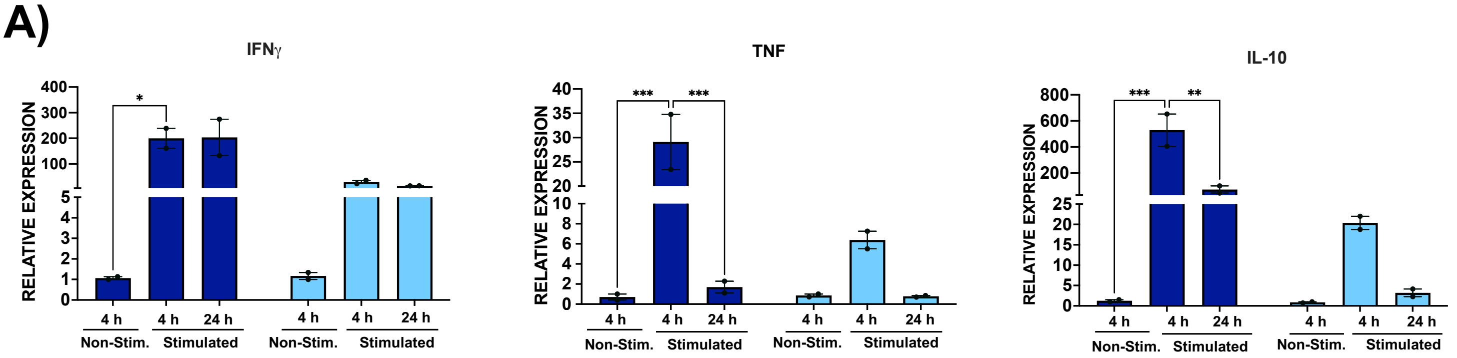

B)

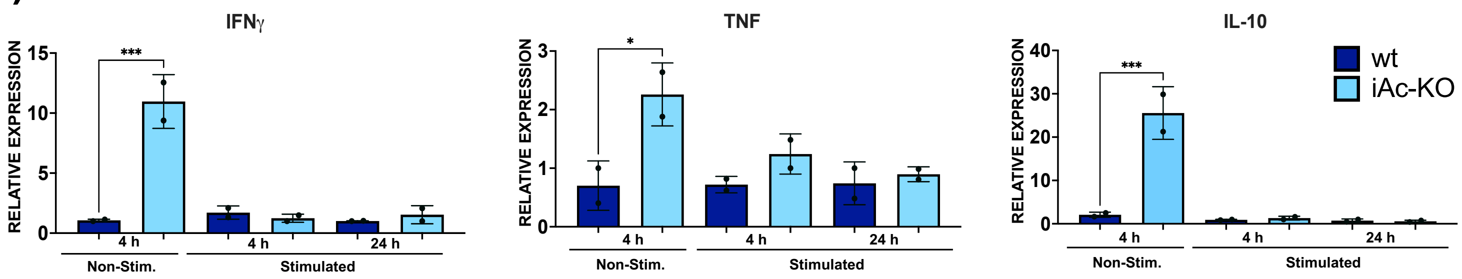

Suppl. Fig. 8. Cytokine mRNA expression profiles of wt and iAc-KO CD4+ T cells after restimulation *in vitro* with T activator beads. **(A)** Time kinetics of mRNA expression levels of the cytokines IFN-γ, IL-10 and TNF were measured in purified wt and iAc-KO CD4+ T cells following 10-day pre-expansion culture and restimulation with T activator beads at a 1:1 ratio for 4 and 24 h. As negative control, nmIg beads were used (Non-Stim.: non stimulated). Statistical analysis was performed using one-way ANOVA with multiple comparisons (*$p<0.05$, **$p<0.01$, ***$p<0.001$). **(B)** For better comparison, expression data were normalized to average mRNA expression by wt CD4+ T cells. Means of triplicate cultures ± SD are shown. The experiment was repeated with similar results**.** Statistical analysis was performed using two-way ANOVA with multiple comparison (*$p<0.05$, ***$p<0.001$).

Suppl. Tab. 1. List of anti mouse antibodies used for flow cytometry

Marker	Clone	Dye	Manufacturer
CD4	RM4-5	Pacific Blue, Alexa Fluor 647	BioLegend
CD8b	YTS156.7.7	PE/Dazzle 594, Alexa Fluor 700	BioLegend
CD25	PC61	PE, FITC	BioLegend
CD44	IM7	FITC	BD Pharmingen
Foxp3	FJK-16S	PE-Cy5	eBioscience
Helios	22F6	FITC, Alexa Fluor 647	Biolegend
Gr-1	RB6-8C5	PE	BD Pharmingen
CD11b	M1/70	FITC	BD Pharmingen
CD3	145-2C11	FITC, Per.CP	BioLegend
NK1.1	PK136	Alexa Fluor 647	BioLegend
IgD	11-26c.2a	FITC	BD Pharmingen
IgM	R6-60.2	PE-Cy7	BD Pharmingen
B220	RA3-6B2	Alexa Fluor 647	BD Pharmingen
CD138	281-2	PE	BD Pharmingen
CD127	SB/199	PE	BioLegend
CD62L	MEL-14	PE-Cy5	BioLegend
KLRG1	2F1/KLRG1	PE-Cy7	BioLegend
CD69	H1.2F3	PE	BioLegend
Ki-67	B56	Alexa Fluor 647	BD Pharmingen
IFNγ	XMG1.2	Alexa Fluor 488	BioLegend
IL-2	JES6-5H4	APC	BioLegend
IL-10	JES5-16E3	PE	BioLegend

Suppl. Tab. 2: HPLC-MS/MS parameter for detection of cellular sphingolipids

Group	Compound	Precursor ion (*m/z*)	Product ion (*m/z*)[a]	Retention time (min)	ISTD
**LCB**	Sph	300.3	**282.3 (8)** / 252.3 (16)	5.5	d_7-Sph
**Cer**	C16:0 Cer	520.5	**264.3 (24)** / 282.3 (24)	13.7	C17:0 Cer
	C18:0 Cer	548.5	**264.2 (24)** / 282.3 (28)	15.6	C17:0 Cer
	C20:0 Cer	576.6	**264.3 (32)** / 282.3 (28)	18.0	C17:0 Cer
	C22:0 Cer	604.6	**264.3 (34)** / 282.3 (30)	21.0	C17:0 Cer
	C24:0 Cer	632.6	**264.3 (36)** / 282.3 (28)	24.5	C17:0 Cer
	C24:1 Cer	630.6	**264.3 (36)** / 282.3 (32)	21.2	C17:0 Cer
**SM**	C16:0 SM	703.6	**184.0 (8)** / 86.1 (76)	12.8	d_{31}-C16:0 SM
	C18:0 SM	731.6	**184.0 (28)** / 86.1 (76)	14.7	d_{31}-C16:0 SM
	C20:0 SM	759.6	**184.0 (28)** / 86.1 (78)	17.0	d_{31}-C16:0 SM
	C22:0 SM	787.7	**184.0 (28)** / 86.1 (78)	19.4	d_{31}-C16:0 SM
	C24:0 SM	815.7	**184.0 (28)** / 86.1 (80)	22.7	d_{31}-C16:0 SM
	C24:1 SM	813.7	184.0 (8) / **86.1 (80)**	19.5	d_{31}-C16:0 SM
**ISTD**	d_7-Sph	307.3	**289.3 (8)** / 259.3 (20)	5.5	-
	C17:0 Cer	534.5	**264.3 (24)** / 282.3 (28)	14.6	-
	d_{31}-C16:0 SM	734.6	**184.0 (28)** / 86.1 (76)	12.7	-

[a] Quantifier mass transitions are given in bold style. Collision energies (in eV) are given in parentheses.

Cer, ceramide; ISTD, internal standard; LCB, long-chain base; Sph, sphingosine, SM, sphingomyelin
